# Prevalence of celiac disease in Moroccan children with type 1 diabetes mellitus: A 16-year cross-sectional study

**DOI:** 10.5339/qmj.2023.37

**Published:** 2024-01-06

**Authors:** Ouijdane Belhiba, Ahmed Aziz Bousfiha, Farida Jennane

**Affiliations:** ^1^Laboratory of Clinical Immunology, Inflammation and Allergy LICIA, Faculty of Medicine and Pharmacy, King Hassan II University, Casablanca, Morocco Email: ouijdanebelhiba@gmail.com ORCID iD: 0000-0002-6523-3177; ^2^Department of pediatric infectious and immunological diseases, Abderrahim El Harouchi Children Hospital, University Hospital Center Ibn Rochd, Casablanca, Morocco; ^3^Pediatric Endocrinology Unit, Abderrahim Harouchi Children’s Hospital, Ibn Rochd Hospital, Casablanca, Morocco

**Keywords:** Celiac disease, type 1 diabetes mellitus, prevalence, children, tissue transglutaminase autoantibodies, Morocco

## Abstract

Background: There is limited data available regarding the prevalence of celiac disease (CD) among children with type 1 diabetes mellitus (T1DM) in Arab countries and the Middle East. This cross-sectional study has been designed to explore the prevalence of CD specifically within the population of Moroccan children and adolescents diagnosed with type 1 diabetes mellitus (T1DM).

Patients and Methods: This is a cross-sectional study of patients who underwent regular follow-up for T1DM at the Pediatric Endocrinology Unit, Abderrahim Harouchi Children’s University Hospital in Casablanca, over a 16-year period from 2004 to 2020. Patients were screened for CD by measuring anti-tissue transglutaminase IgA, and those with positive antibodies underwent endoscopy assessment.

Results and Discussion: All 550 patients regularly followed up with TIDM were screened for CD. Fifty-five (33 girls/22 boys) of the screened patients had histologically documented CD, yielding a prevalence of 10%. Nineteen (41.9%) patients had developed CD within the initial four years of diagnosis with T1DM. Therefore, among the six confirmed CD patients, the average age at the onset of T1DM was 3.7 years. For twenty-four (57.5%) of the patients, exhibited no apparent clinical indications of CD, and their condition was only identified through systematic screening.

Conclusion: This study showed a high prevalence rate of CD associated with type 1 diabetes T1DM, particularly among young children. The results of this paper indicate one of the highest prevalence rates reported in the existing literature for the coexistence of CD and T1DM. These findings may suggest the necessity of a systematic screening of CD in T1DM patients.

## Background

Celiac disease (CD) and type 1 diabetes mellitus (T1DM) are two chronic autoimmune disorders that share common genetic and environmental risk factors. Studies indicate that individuals with CD are more likely to develop T1DM, and vice versa.^1^ The coexistence of celiac disease (CD) and type 1 diabetes mellitus (T1DM) has long been associated with the presence of common high-risk human leukocyte antigen (HLA) genotypes, primarily HLA-DQ2 and/or DQ8.^2^ These genetic factors were thought to be central to the development of both diseases. However, recent research has challenged this notion, suggesting that environmental or non-genetic factors play a substantial role in the association between CD and T1DM.^3^ In addition, the temporal relationship between the two conditions remains a topic of the ongoing investigation. Some children are diagnosed with T1DM before developing CD, while in others the sequence is reversed, suggesting that the interplay between genetic, environmental, and immunological factors in the pathogenesis of these diseases is far from being fully understood.^4^

Type 1 diabetes is an organ-specific autoimmune disorder caused by the immune destruction of β cells, leading to insulin deficiency and entire dependence on exogenously administered insulin.^5^ CD is an immune-mediated enteropathy characterized by small intestinal lesions of variable severity. It may have various symptoms depending on the intestinal lesion; ranging from the asymptomatic form (silent) to the classical form (patent).^6,7^ The most common type is classic or typical CD, characterized by the classic features of intestinal malabsorption and fully developed gluten-induced villous atrophy. People with the classic form of the disease often present gastrointestinal symptoms such as diarrhea, abdominal pain, and weight loss.^8^

Several studies have examined the prevalence of CD in individuals with T1DM, and findings consistently indicate a higher occurrence of CD in the T1DM population compared to the general population. The prevalence of CD in T1DM ranges from 1% to 10%, whereas in the general population, it is estimated to be between 0.3% and 1%.^9,10^ Nonetheless, available data about the prevalence of CD in children with T1DM, especially in Arab countries and particularly within North Africa, like Morocco, remains relatively scarce. Some reports have documented the prevalence of CD in Moroccan children with T1DM.^11^

The presence of coexisting CD within the group of patients diagnosed with T1DM signifies the existence of a distinct subgroup that may face an elevated susceptibility to several significant health concerns. Research has indicated a notable association between CD and heightened risks of hypoglycemia,^12^ retinopathy and nephropathy,^13^ and overall mortality^14^ in individuals with T1DM. Consequently, in light of these findings, a consensus has emerged among various medical guidelines, advocating for a systematic and comprehensive screening for celiac disease in individuals diagnosed with type 1 diabetes.^15,16^

However, despite the advent of sensitive and specific serologic testing, routine screening for CD in diabetic populations is not a universal practice, especially in resource limited settings. In light of these gaps in knowledge and clinical practice, and due to the lack of data on the prevalence of CD in children and adolescents with T1DM, this retrospective cross-sectional study aimed to analyze data from a comprehensive 16-year follow-up period (from 2004 to 2020) and determine the prevalence rate of coeliac disease in Moroccan children and adolescents with T1DM. Given the severe complications associated with the coexistence of these two conditions, especially in this young population, this study aimed to bridge the existing knowledge gap and provide crucial data to guide future clinical practices in managing these complex medical conditions in young patients.

## Methods

### Study design and setting

This retrospective cross-sectional study was conducted on children and adolescents with T1DM, followed up at the Pediatric Endocrinology Unit at Abderrahim Harouchi Children’s University Hospital in Casablanca over 16 years from January 2004 to January 2020.

Between 2004 and 2015, screening was carried out on a minority of patients. Since 2015, the screening of CD became systematic, as part of an annual complication and autoimmune disease screening policy.

Patient data were obtained by questionnaire and included age, gender, time of onset of diabetes, time of the screening of CD, age diagnosis of CD, clinical and auxiological manifestations, family history, biological tests: notably the examination of Anti-Tissue Transglutaminase Antibody (anti-tTGA) levels, presence of other autoimmune diseases, or any acute and chronic complications, and intestinal histopathological findings for CD patients.

### Patients

Eligibility criteria included 550 children and adolescents, aged from 1 to 18 years at the time of diagnosis, regularly monitored for T1DM, and willing to provide informed consent.

All patients with T1DM were diagnosed according to the clinical and biological International Society for Pediatric and Adolescent Diabetes (ISPAD).^17^ The diagnosis of CD was based on European Society for Pediatric Gastroenterology Hepatology and Nutrition (ESPGHAN) guidelines; the patients underwent immunologic testing initially based on IgA anti-tTGA screening. All patients who tested positive for anti-tTGA underwent a second assays after 6 months, as a last step those who had a positive serology were confirmed by duodenal biopsy.^10,16^ The diagnoses of all the diseases such as thyroiditis, Down syndrome, nephropathy, and child growth retardation have been developed based on international health criteria established by globally recognized medical and scientific institutions, including the World Health Organization (WHO).

### Laboratory testing methods

All patients underwent screening using serum anti-tissue transglutaminase antibodies (anti-tTGA), which were determined through an enzyme-linked immunosorbent assay (ELISA) utilizing human recombinant tTGA as the antigen. Patients displaying positive anti-tTGA results proceeded to undergo a confirmation process for CD through upper gastrointestinal endoscopy, accompanied by intestinal biopsies. Multiple biopsies were extracted from both the second portion of the duodenum (4-6 biopsies) and the duodenal bulb (2 biopsies). The biopsy specimens were then meticulously examined by an expert pathologist. Histology showing an “infiltrative lesion” with intraepithelial lymphocytosis was considered Marsh I, an “infiltrative-hyperplastic lesion” as Marsh II, and “villous atrophy” in addition as Marsh III (partial-IIIa, subtotal-IIIb, total-IIIc). The presence of histological patterns consistent with CD, classified according to the Marsh-Oberhuber classification^18^ (Marsh 1-3 lesions), was deemed indicative of CD diagnosis, particularly in conjunction with positive CD antibody results.

### Statistical Analyses

SPSS version 20.0 was used for the statistical analysis; Continuous variables were represented as mean ± standard deviation (SD), and categorical variables as percentages. Chi-square test exact tests were used to observe an association between the qualitative study and outcome variables. All p-values < 0.05 were considered statistically significant for individual variables.

### Ethical Considerations

In September 2019, the Ethics Committee of the Faculty of Medicine and Pharmacy Hassan II University approved the study under reference number 14/19, and written consent was obtained from the children’s parents or guardians after describing the study in detail.

## Results

### Participants’ Characteristics

This retrospective cross-sectional study was conducted in the Pediatric Endocrinology Unit, at Abderrahim Harouchi Children’s University Hospital in Casablanca. The cohort included 550 children (259 girls and 291 boys) ranging in age from 1 to 18 years at the time of their T1DM diagnosis. The mean age of the participants was 13.7 ± 5.7 years, with a range of 1 to 34 years. The average age of T1DM diagnosis was 5.9 ± 3.9 years. The demographic results of this study are presented in Table 1. A predominance of females in the CD+ group was observed, accounting for 60% of the cases. Furthermore, the mean duration of T1DM among the participants was 7 ± 4.9 years.

### Serologic and Histological Screening

Among 550 patients who underwent anti-tTGA tests, 68 initially tested positive. After six months, a follow-up test was conducted, revealing that only 55 patients still had positive results. All 55 patients underwent histological confirmation of celiac disease (CD), resulting in a prevalence rate of 10%. Among the cohort of 55 individuals who tested positive for anti-tTGA, a substantial majority, accounting for 83%, exhibited distinct histological features indicative of villous atrophy. Among these patients, the distribution of villous atrophy subcategories included 6 cases of partial villous atrophy, 17 cases of subtotal villous atrophy, and 23 cases of total villous atrophy. Additionally, 4 patients demonstrated an infiltrative-hyperplastic lesion pattern, while 5 patients displayed an infiltrative lesion profile.

The mean age at the time of CD diagnosis was 6.4 ± 4.5 years, ranging from 1 to 18 years.

Out of the 55 cases, 23 (57.5%) were identified through systematic screening (silent form) without any noticeable clinical manifestations, while the remaining patients had already presented with some clinical symptoms.

Five patients had a first-degree relative with only CD and six patients with only T1DM. A significant gender difference (p value=0.043) was observed between the two groups, with a higher proportion of males in the group without CD (52.9%), and a predominance of females in the group with CD (60%).

**Figure 1.**
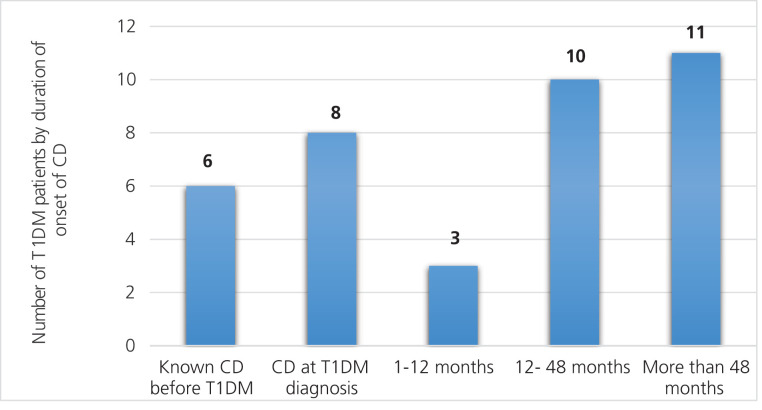
The onset of T1DM in relation to the onset of CD. *Abbreviations*: T1DM: Type 1 diabetes mellitus; CD: Celiac disease.

Among the children, six (13.9%) had a pre-existing CD diagnosis before T1DM onset. At the onset of TIDM, eight children (18.6%) had CD, and 13 (30.3%) of the children had developed CD between 12 months and 48 months after clinical diagnosis with a T1DM. Furthermore, eleven T1DM children (25.5%) developed CD more than 48 months after the T1DM diagnosis (Figure 1). Out of the 55 patients included in the study, 17 individuals were excluded from the analysis due to incomplete or missing information.

### CD and other Associated Diseases

Within the cohort of 55 patients diagnosed with CD in this study, it was observed that some individuals presented with additional conditions other than T1DM. Among these patients, three individuals had immune thyroid disease, specifically two cases of hypothyroidism and one case of hyperthyroidism. Additionally, one subject with Down syndrome, one with nephropathy related to diabetes, and two with growth retardation were identified. These findings highlight the presence of coexisting medical conditions in individuals with CD, emphasizing the importance of comprehensive medical assessment and management to address the various health needs of these patients.

## Discussion

T1DM is one of the autoimmune disorders affecting the general population, and particularly children and adolescents. This disease is associated with several other autoimmune diseases, the most common ones being hypothyroidism, CD, and Addison’s disease.^6^

The prevalence of CD in the general population has been reported to be 1%, while the prevalence of CD in patients with T1DM ranges between 1 and 10%.^2^ The variation in the prevalence of celiac in the literature is due to the different racial/ethnic populations studied and the method used to confirm diagnosis.

The prevalence of celiac disease was evaluated based on histology rather than positive antibody markers, in a cohort of 550 children followed-up for T1DM.

In Morocco, data on CD associated with T1DM prevalence in Morocco is limited, with few reports documenting the prevalence of CD in Moroccan children.^11^

The prevalence of biopsy-proven CD among Moroccan children with T1DM (10 %) is lower than that in the Arab population in the Middle East: Saudi Arabia 19.7%,^19^ Oman 17%,^20^ but is comparable to the North African Arabs: Libya 11%,^21^ Algeria 16%;^22^ and Europeans: Sweden 9.1%,^23^ Spain 13.4%^24^ (Table 2).

The findings of this paper align with existing literature, which indicates that celiac disease (CD) often emerges within the first four years after the diagnosis of type 1 diabetes mellitus (T1DM). This concurrence is particularly salient in children with T1DM who simultaneously develop CD, where the onset of diabetes tends to occur at an earlier age, typically between 3.2 and 5.7 years old, in comparison to their counterparts with other associated autoimmune disorders.^15,32^

Consistent with these findings, the results of this study show that out of the 55 patients with CD, 21 individuals (55%) developed CD within the first four years after their T1DM diagnosis, with a median age of 5 years (ranging from 1 to 13 years). Additionally, among the six patients who had CD before the onset of T1DM, the mean age at CD development was 3.7 years (ranging from 1 to 8 years).

Furthermore, in line with previous research, it is confirmed that the majority of children in this study developed T1DM before CD. Specifically, 24 patients (63%) had T1DM preceding the diagnosis of CD.^32^ This finding reinforces the temporal sequence often observed between these two conditions.

In this series, a female predominance (60%) in the CD group is reported, which is consistent with the study by Cerutti et al.^32^ These authors emphasized the impact of sex and age on the risk of developing CD in individuals with T1DM. They concluded that the risk of developing both diseases is higher among females, particularly those diagnosed with diabetes before the age of 4 years.^10^

This study reveals an interesting finding that more than half (57.5%) of the patients diagnosed with CD were identified solely through systematic screening, without experiencing any clinical symptoms. These individuals were classified as having the asymptomatic form of CD. In contrast, the remaining patients were diagnosed after presenting with typical symptoms such as diarrhea, abdominal pain, and weight loss. The identification of a significant proportion of CD cases through systematic screening highlights the importance of proactive case-finding strategies, particularly in high-risk populations like individuals with T1DM. The natural history of silent and potential celiac disease is not yet fully understood, and it remains unclear whether they represent progressive stages of CD or distinct subtypes.^33^ Further research is needed to investigate the progression and outcomes of silent and potential CD, and whether intervention at these early stages can alter the course of the disease.

It is widely acknowledged that a substantial number of CD cases go undiagnosed in the general population. However, implementing a strategy to expand case-finding among high-risk populations, especially individuals with T1DM, can effectively identify these undiagnosed cases. This study highlights the importance of reviewing screening protocols in clinical practice. Healthcare professionals should consider routinely screening children with T1DM for CD, even at a very young age, to enable early detection and appropriate management. Furthermore, in terms of patient education, it is essential to inform parents and caregivers of children with T1DM about the increased risk of CD and the potential benefits of early diagnosis and dietary management.

Aligned with these goals, the findings of this paper have been effectively communicated to the medical practitioners, enabling them to consider potential avenues for further exploration, such as biopsies, and, if deemed necessary, initiation of a gluten-free diet (GFD).

This retrospective study is subject to several limitations, with the primary constraint being the presence of missing or incomplete patient information, which led to their exclusion from the analysis. This can introduce bias into the results and restrict the generalizability of the findings to a wider population. Additionally, some patients may not consistently follow up with their condition, leading to an underestimation of symptoms or complications related to T1DM or celiac disease CD. Furthermore, due to financial constraints, not all patients in the study could undergo genotyping to study the correlation between these two autoimmune diseases.

The findings of this study underscore the necessity for continued research, particularly in the field of genetics. Investigating specific genetic markers or variations that render certain individuals with T1DM more susceptible to developing CD holds immense relevance. By exploring the genetic underpinnings of this coexistence, researchers can potentially identify key factors contributing to the simultaneous occurrence of these conditions.

## Conclusion

In this retrospective study, one of the highest prevalence rates is reported in the literature for CD associated with T1DM, with onset in particularly young children. These findings highlight the significance of implementing broader screening protocols in clinical practice. Also, these findings underscore the advantage of implementing a systematic screening for CD in diabetic patients, hopefully minimizing the potential risk of complications due to undiagnosed CD. By implementing systematic screening, healthcare providers can identify CD at an early stage and ensure appropriate management, ultimately improving the overall health of people with T1DM.

## List of Abbreviations

Anti-tTGA: Anti-tissue transglutaminase Antibody

CD: Celiac Disease

ELISA: Enzyme-Linked Immunosorbent Assay

ESPGHAN: European Society for Paediatric Gastroenterology Hepatology and Nutrition

HLA: Human Leukocyte Antigen

ISPAD: International Society for Pediatric and Adolescent Diabetes

MENA: Middle East and North Africa

SD: Standard Deviation

T1DM: Type 1 Diabetes Mellitus

WHO: World Health Organization.

## Conflicts of Interest

The authors declared that there were no competing interests.

## Funding Sources

This work was not supported by the Fund for Scientific Research.

## Authors’ Contributions

OB led the research and led the writing of the paper. AAB provided critical feedback and helped shape the research, analysis, and manuscript. FJ supervised the research and successive drafts of the paper.

## Acknowledgments

We would like to thank all the participants for their contribution to this study.

## Figures and Tables

**Table 1.** Characteristics of 550 patients of our study.

	Total (550)	Celiac disease		P-value
		Group 1 CD+ (55)	Group 2 CD-(495)	
Age (years)	13.7 ± 5.7	13.3 ± 7.3	13.9 ± 5.5	0.342
Sex female (%)	259 (47.1%)	33 (60%)	226 (41.1%)	0.043*
Age at diagnosis T1DM (years)	5.9 ± 3.9	4.4 ± 3.8	6.1 ± 3.9	0.764
Duration of diabetes (years)	7 ± 4.9	7.9 ± 6.5	6.9 ± 4.7	0.293

Mean ± SD

*Note*: Bold values are statistically significant, p < 0.05.

*Abbreviations*: T1DM: Type 1 diabetes mellitus; CD: Celiac disease; CD+ = Presence of celiac disease; CD- = Absence of celiac disease.

**Table 2.** Prevalence data for celiac disease in patients with type 1 diabetes in different continents.

Geographical origin	Country (N)	Serological prevalence (n)	Period of the study	References
North Africa	Morocco (N=550)	10% (55)	2004-2020	-
	Tunisia (284)	3.52% (24)	1981-1994	[25]
	Algeria (116)	16% (13)	1993-1994	[22]
	Libya (218)	11% (24)	2008	[21]
Europe	Italy (4379)	4.22% (185)	2007-2011	[26]
	Sweden (847)	9.1% (77)	1995-2004	[23]
	France (370)	3.2% (9)	2001-2002	[27]
	Spain (463)	13.4% (62)	-	[24]
Asia	Iran (83)	14.4%(13)	-	[28]
	Turkey (38)	7.8% (3)	-	[29]
	India (103)	13.6% (14)	-	[30]
	Oman (93)	17% (16)	2011-2012	[20]
	Saudi Arabia (228)	19.7% (45)	2013-2014	[19]
Australia	Australia (4379)	4.22% (185)	1990-2009	[31]
North America	North of America (158)	7% (11)	-	[9]

*Note:* N: number of cases studied; n: number of patients with CD.
